# Toxin ζ Reversible Induces Dormancy and Reduces the UDP-*N*-Acetylglucosamine Pool as One of the Protective Responses to Cope with Stress

**DOI:** 10.3390/toxins6092787

**Published:** 2014-09-18

**Authors:** Mariangela Tabone, Silvia Ayora, Juan C. Alonso

**Affiliations:** Department of Microbial Biotechnology, Centro Nacional de Biotecnología, CNB-CSIC, C/Darwin 3, Madrid 28049, Spain; E-Mails: mariangela.tabone@cnb.csic.es (M.T.); sayora@cnb.csic.es (S.A.)

**Keywords:** toxin-antitoxin system, fosfomycin, vancomycin, ampicillin, bacterial persistence

## Abstract

Toxins of the ζ/PezT family, found in the genome of major human pathogens, phosphorylate the peptidoglycan precursor uridine diphosphate-*N*-acetylglucosamine (UNAG) leading to unreactive UNAG-3P. Transient over-expression of a PezT variant impairs cell wall biosynthesis and triggers autolysis in *Escherichia coli*. Conversely, physiological levels of ζ reversibly induce dormancy produce a sub-fraction of membrane-compromised cells, and a minor subpopulation of *Bacillus subtilis* cells become tolerant of toxin action. We report here that purified ζ is a strong UNAG-dependent ATPase, being GTP a lower competitor. *In vitro*, ζ toxin phosphorylates a fraction of UNAG. *In vivo*, ζ-mediated inactivation of UNAG by phosphorylation does not deplete the active UNAG pool, because expression of the toxin enhances the efficacy of genuine cell wall inhibitors (fosfomycin, vancomycin or ampicillin). Transient ζ expression together with fosfomycin treatment halt cell proliferation, but ε_2_ antitoxin expression facilitates the exit of ζ-induced dormancy, suggesting that there is sufficient UNAG for growth. We propose that ζ induces diverse cellular responses to cope with stress, being the reduction of the UNAG pool one among them. If the action of ζ is not inhibited, e.g., by *de novo* ε_2_ antitoxin synthesis, the toxin markedly enhances the efficacy of antimicrobial treatment without massive autolysis in Firmicutes.

## 1. Introduction

Microbial type II toxin-antitoxin (TA) systems, which are stress regulators, are generally composed of two genes organized in an operon, encoding a toxin (T) and an antitoxin (A) protein [1,2,3]. RelE and ζ are the most abundant prokaryotic toxins in nature [4], with ζ been the most ubiquitous toxin in Firmicutes [5,6]. The plasmid- or chromosomal-encoded TA complexes (ζ-ε or PezT-PezA) consist of two monomeric long-living ζ (PezT) toxins separated by a dimeric short-living ε_2_ (PezA_2_) antitoxin, forming a ζε_2_ζ (PezTPezA_2_PezT) inactive complex [7,8]. The toxins of the ζ family are highly conserved, ζ (286 amino acids long polypeptide) shares 42% of sequence identity with PezT (253 amino acids long). Both toxins have a very similar three-dimensional structure, and mutations in equivalent positions show similar phenotypes [7,8]. However, antitoxin ε (90 amino acids long polypeptide) only shares 17% of sequence identity with the C-terminal region of antitoxin PezA (158 amino acids long). Despite this low sequence identity, the C-terminal region of PezA folds into a three helix bundle similar to that of ε [7,8].

Both Firmicutes toxins, ζ and PezT might interact with ATP-Mg^2+^ or GTP-Mg^2+^, with uridine diphosphate-*N*-acetylglucosamine (UNAG) and with ε_2_ (PezA) antitoxin [7,9]. These toxins, as part of the biological non-toxic heterotetrameric (ζε_2_ζ or PezTPezA_2_PezT) complex, interact with UNAG [9]. However, in this inactive complex, the toxin cannot interact with ATP-Mg^2+^/GTP-Mg^2+^, because the antitoxin helixes block the entry of the nucleotide cofactor into the binding pocket of the toxin through steric hindrance [7,8,10]. Toxin PezT phosphorylates the 3'-OH group, which is an essential precursor of bacterial cell wall biosynthesis, leading to phosphorylated, unreactive, UNAG-3P [9]. Toxin PezT was specific for UNAG, because it failed to phosphorylate uridine diphosphate-*N*-acetylgalactosamine [9]. Hence, ζ /PezT toxin blocks the first committed step in the peptidoglycan synthesis pathway, that is the transfer of an enolpyruvate residue from phosphoenolpyruvate to position 3 of UNAG [9].

Toxin ζ, under various stress stimuli, can be activated as a result of ε_2_ antitoxin degradation by stress-induced proteases or under conditions that prevent TA gene expression [8,11,12,13]. Such activation permits free wt ζ (or its short-living variant (ζY83C, half-life ~28 min)) to trigger a heterogeneous set of protective responses, and the expression of ~2% of total *Escherichia coli* or *Bacillus subtilis* genes, so that these toxins show a bacteriostatic, rather than a bactericidal behavior [12,14]. In *B. subtilis* cells, ζY83C toxin expression increases RelA expression, reduces expression of lipid metabolism genes, and decreases the GTP pool without apparent alteration of the proteome within the first 15 min upon induction [12,14,15]. Within the 15–60 min interval, ζY83C expression reduces the synthesis of macromolecules, decreases ATP levels, increases the alarmone guanosine (penta)tetraphosphate ([p] ppGpp) pool, and alters the membrane potential without affecting the SOS response [14,15]. Within 60–90 min, ζY83C expression decreases the pool of a novel metabolite, probably UNAG [14]. Finally, expression of ζ for 120–240 min leads to a fraction (25%–35%) of the population stained with propidium iodide (PI), suggesting a membrane compromise; but subsequent expression of the ε_2_ antitoxin facilitates the exit from the dormant state and a fraction of cells recover their membrane “alteration” [12,14,15]. A different effect is observed if *B. subtilis* cells express the wt ζ or ζY83C toxin for longer periods of time (>960 min). Here, the cells enter in a point of no return, and ε_2_ antitoxin expression cannot reverse the dormant state [12,16]. Conversely, in *E. coli* cells PezT over-expression inhibits cell wall synthesis and triggers autolysis 60 min after induction [9]. It is likely that toxin concentrations or time of exposure shows hormesis, complicating the understanding of ζ toxin mode of action.

### 1.1. Aims of the Experiments

Previously, it was been proposed that a PezT variant lacking the C-terminus (PezTΔC242) instigates a suicide program with subsequent autolysis [9], but ζ works as a checkpoint barrier inducing a reversible dormant state without massive cell lysis [14]. In the first outcome, PezT toxin may phosphorylate all the UNAG pool and by this way may provoke a cell membrane compromise and lysis, whereas ζ toxin acts on different cellular processes, not only cell wall synthesis. In order to discriminate between these two hypotheses, different experimental approaches were used in this report. First, ζ was purified, free of ε_2_ antitoxin, to test whether ATP hydrolysis is coupled or not with the phosphate transfer to UNAG. In addition, we analyzed whether GTP can be used as a donor of the phosphate group. Second, we tested whether ζ expression depletes the UNAG pool or only reduces it. For that purpose, we used antimicrobials that inhibit cell proliferation by targeting cell wall synthesis with fosfomycin (Fos), vancomycin (Van), ampicillin (Amp) upon transient expression of the ζY83C toxin variant in *B. subtilis* cells. Third, we studied if upon specific reversal of ζ-induced dormancy *B. subtilis* cell proliferation is recovered or there is cell lysis. To perform the latter study ζ was transiently expressed in the presence or absence of Fos, and then ε_2_ antitoxin was induced. Finally, we tested whether ζ expression for long periods of time causes cell lysis in *B. subtilis*.

## 2. Results and Discussion

### 2.1. Toxin ζ Hydrolyses ATP in the Presence of UNAG

Recently, it was documented that in the presence of ATP-Mg^2+^, ζ toxin, in the presence of ε_2_ antitoxin, phosphorylates the cell wall precursor UNAG by attaching a phosphoryl group to the 3'-hydroxyl group of the *N*-acetylglucosamine moiety to form UDP-*N*-acetylglucosamine-3'-phosphate (UNAG-3P) [9]. The low efficiency of this reaction, which was not stoichiometric, could be attributed to the presence of the antitoxin or because the phosphorylation reaction might be uncoupled from the ATPase reaction. In this work we have we tested whether ζ toxin purified by a novel protocol, free of antitoxin (as described in the Experimental section), is able to hydrolyze ATP in the presence or absence of UNAG. We have also tested whether the hydrolysis reaction is coupled with the phosphotransfer reaction and if GTP could work as a donor of inorganic phosphate (P_i_) for the transfer reaction. The products were separated from the substrate by thin-layer chromatography (TLC) and analyzed by autoradiography.

In the presence of UNAG, ζ toxin hydrolyzed [γ^32^P]-ATP, and we observed the accumulation of a radioactive product(s) which co-migrate with the front (data not shown). This was the expected position for the product of ATP hydrolysis, which is inorganic phosphate [^32^Pi]. We do not know how [γ^32^P]-UNAG-3P should migrate under the conditions used, but one hypothesis is that it co-migrates with the TLC front under the experimental conditions used. To address this, we perform experiments with other TLC eluents in order to separate [^32^Pi] from [^32^P]-UNAG-P, but we failed to have a significant separation of both of them under the conditions used (data not shown). Alternatively, the reaction is uncoupled and [γ^32^P]-UNAG-P accounts only to a minor fraction of the radiolabelled product.

**Figure 1:**
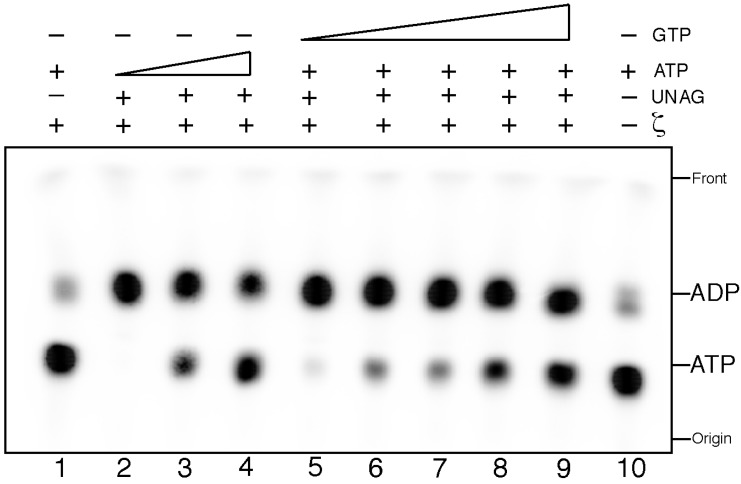
Uridine diphosphate-*N*-acetylglucosamine (UNAG)-dependent ζ hydrolysis of ATP is poorly competed by GTP. Samples containing 2 mM UNAG and increasing concentrations of ATP (0.5, 5 and 10 mM (with a fixed concentration of α^32^P-ATP, 10 nM), lanes 2–4) or 2 mM UNAG and a fixed concentration of ATP (0.5 mM [α^32^P-ATP, 10 nM]) and increasing GTP concentrations (1.25, 2.5, 5, 7.5 and 10 mM, lanes 5–9) were incubated with 0.5 µM ζ toxin for 30 min at 30 °C in buffer B. ATP hydrolysis was analyzed by thin-layer chromatography (TLC) performed on polyethyleneimine-cellulose plates with 0.85 M KH_2_PO_4_ (pH 3.4) as the mobile phase, followed by autoradiography.

To quantify the rate of ATP hydrolysis the experiments were performed using [α^32^P]-ATP. If ζ hydrolyses [α^32^P]-ATP it should be converted onto [α^32^P]-ADP that runs faster than [α^32^P]-ATP in a TLC. The commercial radioactive [α^32^P]-ATP contains 6%–10% of [α^32^P]-ADP [17], hence this is our background level, and this was used in the TLC as an internal control that marked the respective running positions of substrates and products. In the absence of ζ toxin no [α^32^P]-ATP hydrolysis was observed (Figure 1, lane 10). Purified ζ toxin (0.5 µM) did not hydrolyze [α^32^P]-ATP (0.5 mM) in the absence of UNAG, but ζ toxin hydrolyzed >85% of the ATP substrate upon addition of UNAG (2 mM), in a 60 min reaction (Figure 1, lanes 1 and 2).

When saturating ATP concentrations were used, also a large fraction of the ATP was hydrolyzed (Figure 1, lanes 3 and 4). At ATP:UNAG ratios of 2.5:1 or 5:1 ~70% and ~40% of radiolabelled [α^32^P]-ATP was converted onto [α^32^P]-ADP, respectively, in a 60 min reaction (Figure 1, lanes 3 and 4). Since the conversion of ATP onto ADP was significantly higher than if each P_i_ generated was transferred to the 3'-OH group of the sugar moiety of UNAG, we tentatively assume that the reaction is not tightly coupled. Previously, it was shown that ζ toxin, in the presence of ATP-Mg^2+^ and the ε_2_ antitoxin, attaches a phosphoryl group to the 3'-hydroxyl group of the *N*-acetylglucosamine moiety to form UDP-*N*-acetylglucosamine-3'-phosphate (UNAG-3P) [9]. Hence we assume that ζ toxin alone (free of ε_2_ antitoxin) would also transfer the P_i_ to form UNAG-3P, but it remains to be shown whether the 3'-OH group of the sugar moiety of UNAG is the only target of the phosphorylation reaction.

To verify the structurally suggested preference for ATP *vs.* GTP (as reported for other phosphotransferases) as substrate, we performed competition experiments. Here, ζ toxin was incubated with increasing ATP or GTP concentrations containing a fixed amount of ATP (0.5 mM ATP containing [α-^32^P]-ATP 10 nM) and UNAG (2 mM). Toxin ζ can hydrolyze ATP with similar efficiency in the presence of a 2.5-fold excess of GTP (Figure 1, lane 5). GTP:ATP ratios of 5:1 to 10:1, marginally decreased ζ-mediated [α^32^P]-ATP hydrolysis (Figure 1, lanes 6 and 7). At GTP:ATP ratios of 15:1 and 20.5:1, ~37 and ~46% of [α ^32^P]-ATP was not converted to [α^32^P]-ADP, respectively (Figure 1, lanes 8 and 9). It is likely therefore that: (I) ζ toxin is preferentially a UNAG-dependent ATPase; (II) the phosphotransfer reaction might not simply be a one-step coupled reaction; and (III) ζ toxin preferentially hydrolyzed ATP-Mg^2+^ over GTP-Mg^2+^ in the presence of UNAG.

### 2.2. Toxin ζ Phosphorylates a Fraction of UNAG In Vitro

In the previous section was shown that ζ requires the presence of UNAG to hydrolyze ATP, suggesting that UNAG or UNAG-3P might stimulate ζ-mediated ATP hydrolysis. To further analyze this, UNAG and ATP were incubated in the absence or presence ζ and the products of the reaction were analyzed by mass spectrometry as described in the Experimental section.

**Figure 2:**
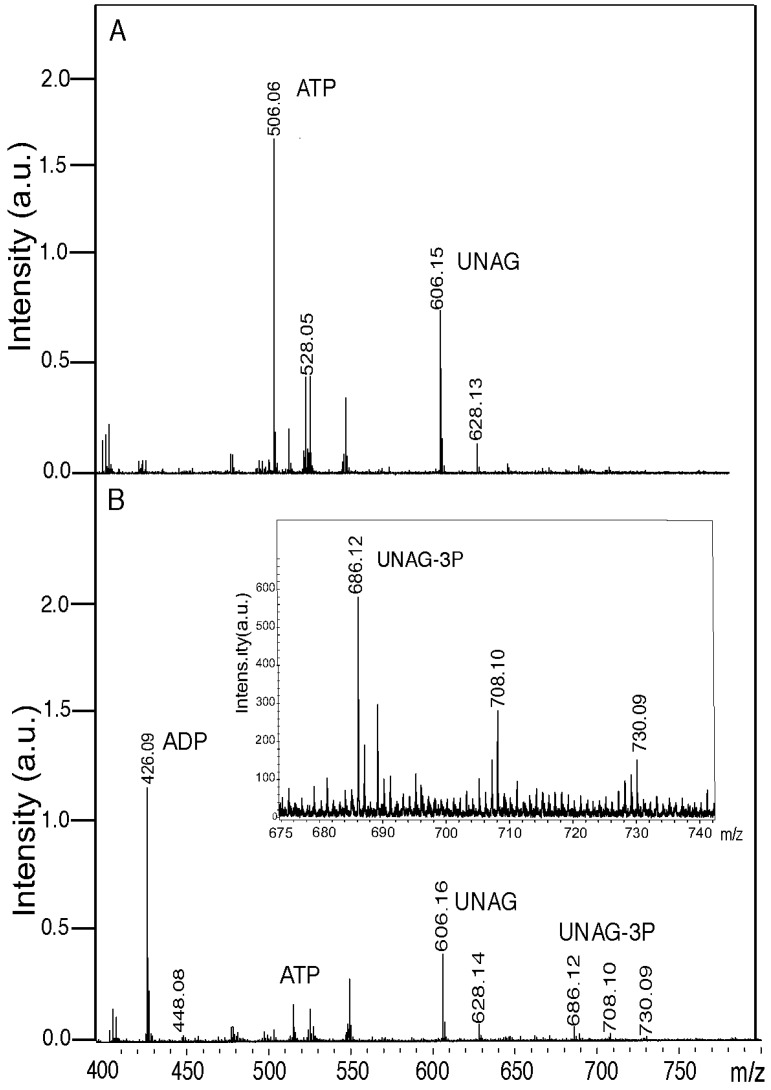
ζ toxin phosphorylates *in vitro* a fraction of UNAG. Samples containing 2 mM UNAG and 0.5 mM ATP were incubated in the absence (**A**) or presence (**B**) of ζ toxin (0.5 µM) for 60 min at 30 °C in buffer B. The reaction products were analyzed by mass spectroscopy. The peaks corresponding to relevant products (ATP, ADP, UNAG and UNAG-3P) are indicated. The intensity (×10^4^) was expressed in arbitrary units (a.u.). In panel B, the 675 to 740 *m*/*z* section is enlarged in the insert.

In a mock reaction, lacking the ζ toxin, phosphorylated UNAG was not observed (compare Figure 2A,B). In all conditions the nucleotide (or compound) and its Na-bound (e.g., M-H^+^ [ATP, 506.06 peak], the M-2H^+^-Na^+^ [ATP-Na_2_, 528.05 peak] or even M-3H^+^-2Na^+^ forms (due to the contribution of the buffer used) were detected (Figure 2A). In the presence of ζ toxin, a massive degradation of the ATP pool (506.06 Da peak, expected 507.18 Da) with subsequent accumulation of ADP (426.06 Da peak, expected 427.20 Da) was observed. However, the proportion of UNAG (606.15 Da UNAG peak, expected 607.35 Da) converted into a 687.35 Da product (UNAG-3P) with a small peak at 686.12 Da (UNAG-3P-Na_2_) was detectable but poor (Figure 2). A parallel TLC analysis of this reaction revealed that ζ toxin hydrolyzed more than 95% of the ATP substrate (data not shown).

As revealed in Figure 2B, in the presence of ζ toxin traces of the ATP pool and a reduced fraction of UNAG were detected by mass spectrometry, when compared to the absence of ζ toxin (Figure 2A). Since traces of ATP and a significant fraction of UNAG remained after 30 min reaction, it was assumed, as done in the previous section (Figure 1), that only a fraction of the P_i_ was transferred to UNAG to generate UNAG-3P (Figure 2B). Alternatively, ζ toxin phosphorylates UNAG in a coupled reaction, but under the conditions used we are loosing a fraction of the accumulated inactive UNAG-3P product.

### 2.3. Toxin ζY83C Leaves Significant Amounts of UNAG In Vivo

*In vitro* assays showed that ζ is a poor kinase, phosphorylating only a fraction of the UNAG present. However, these assays might not extrapolate to the *in vivo* situation. It could be that *in vivo* there is(are) some unknown factor(s) that may stimulate the phosphotransfer reaction. To test whether ζ toxin reduces or depletes the UNAG pool *in vivo*, and by this way it reduces or blocks the biosynthetic pathway of peptidoglycans different types of antimicrobials, which act at different levels of the two stage process of cell wall biosynthesis, were used.

It has been established that the first step of peptidoglycan (murein) biosynthesis is a cytosolic stage that consists in the transfer of an enolpyruvate moiety from phosphoenolpyruvate (PEP) to the C3 position of *N*-acetylglucosamine moiety of UNAG, a reaction catalyzed by two MurA enzymes in Firmicutes (the ubiquitous MurA [MurAA or MurA1] and MurAB [or MurA2] [18]), to yield enolpyruvyl-UDP-*N*-acetylglucosamine (EP-UNAG). The synthesis of these murein precursors can be inhibited by: (I) ζ-mediated phosphorylation of UNAG to form unreactive UNAG-3P; and (II) a naturally occurring PEP analogue, Fosfomycin (Fos), which binds covalently to the active site of MurAA (or MurAB). Fos, in the presence of the UNAG substrate inhibits cell wall biosynthesis by blocking the accumulation of EP-UNAG [19,20]. EP-UNAG is transformed by a series of cytosolic steps, which results in the formation of the UDP-*N*-acetyl-muramic acid-pentapeptide that is translocated to the membrane acceptor and through different steps the *N*-acetylglucosamine/*N*-acetylmuramic acid-pentapeptide is covalently linked to the lipid carrier molecule undecaprenol via a pyrophosphate ester bridge (Lipid II). Vancomycin (Van) prevents incorporation of *N*-acetylglucosamine and *N*-acetylmuramic acid-peptapeptide subunits into the peptidoglycan matrix, and interferes with the Lipid II cycle [21]. Then, the Lipid II building block is used as the substrate for the polymerization reaction, consisting of the assembly into glycan chains by transglycosilation and peptide cross-linking of the pentapeptide moiety by transpeptidation, reactions catalyzed by Classes A or B penicillin-binding proteins [22,23,24]. Amp acts as an irreversible inhibitor of these transpeptidases [25,26].

We reasoned that if ζ toxin depletes the UNAG pool, cells will become tolerant to antimicrobials that act downstream of toxin action, leading to non-inheritable drug tolerance (persistence). To test this hypothesis, ζ toxin induced cells were exposed to Fos, Van or Amp and the antimicrobial drug tolerance was analyzed. These antimicrobials, in addition of inhibiting cell wall synthesis as explained above, induce two regulatory systems to cope with stress in Firmicutes: the extracytoplasmic function (ECF) σ factors and/or cell envelope stress-sensing two component systems, which are linked to other stress responses [27]. Van, which is the strongest cell envelope-perturbing agent, induces the synthesis of a large number of genes controlled by specific σ factors (σ^V^, σ^M^, σ^W^ and σ^Y^) and two components systems (e.g., LiaRS), Fos alters the expression of genes controlled by σ^M^ and σ^W^; and Amp alters the expression of genes through cell envelope stress-sensing two component systems (e.g., BlaRI, MecRI) [27]. If the three antimicrobials show a similar behavior upon ζ toxin we can omit any specific contribution of cell envelop stress.

**Figure 3:**
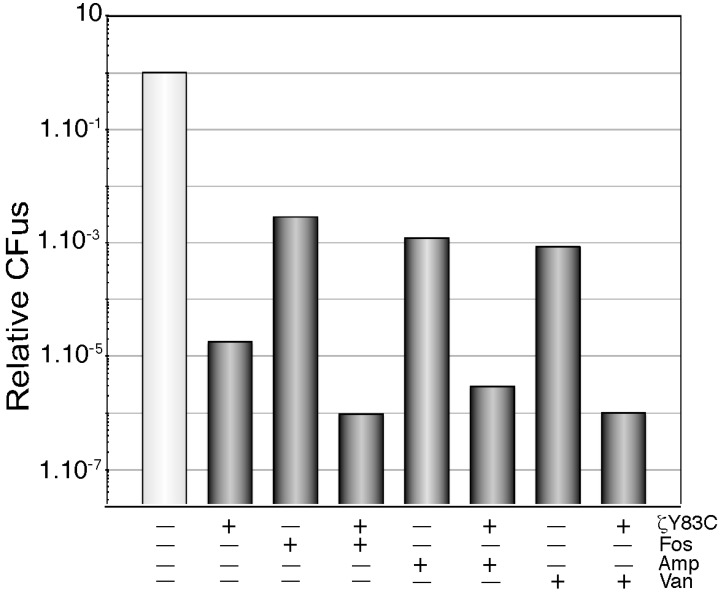
Cell wall inhibitors and ζ toxin expression show an additive effect. BG689 cells containing the short living ζ variant (ζY83C) gene were grown to ~5 × 10^7^ cell/mL in MMS7. Then Xyl (0.5% to induce expression of the ζY83C toxin) or an antimicrobial or both was added and the culture was incubated for 120 min. Cells were washed and appropriate dilutions were plated to count the survivals. The results are the average of at least four independent experiments and are within a 10% standard error.

To perform these experiments we took advantage of the short-lived toxin variant, ζY83C (half-life ~28 min) under control of a Xyl inducible promoter (*xylR P*_XylA_ζY83C expression cassette) (Table 1). The level of toxin in non-induced BG689 cells is too low (<10 ζY83C monomers/colony forming units, CFUs) to measurably alter the growth rate in minimal medium S7 (MMS7) [12]. Previously it was observed that induction of the *xylR P*_XylA_ζY83C cassette (BG689 cells), by addition of 0.5% Xyl, increased ζY83C levels to a plateau with a toxin concentration of ~300 toxin monomers/CFUs at ~10 min, and the steady-state level of the toxin remained for at least 240 min [12]. When exponentially growing BG689 cells, growing in MMS7, reached moderate-density (~5 × 10^7^ cells/mL) xylose (Xyl) 0.5% was added to express ζY83C toxin as described Materials and Methods [14,15]. As already observed, transient exposure to sub-physiological concentrations of free ζY83C induced dormancy and produced a typical biphasic survival curve, with a sub-fraction of cells (1–5 × 10^−5^ cells) non-inheritable tolerant to toxin action upon 120 min of exposure (Figure 3) [14,15].

**Table 1:** Bacterial strains used.

Strains	Relevant genotype	Reference
BG687 ^a^	+*xylR*, *P_xylA_*, *cat*	[12]
BG689 ^a^	*+xylR*, *P_xylA_* ζY83C, *cat*	[12]
BG1127 ^a^	+*lacI*, *P_hsp_*, *spc*, [pCB799, *xylR*, *P_xylA_* ε, *cat*]	[12]
BG1125 ^a,b^	+*lacI*, *P_hsp_* ζ, *spc*, [pCB799, *xylR*, *P_xylA_* ε, *cat*]	[12]
BL21(DE3) ^c^	+[pCB920, *P*_T7_ ζ gene, *P*_ω_ ω and ε genes, *bla*]	This work

^a^ The *B. subtilis* strains are isogenic with BG214 (*trpCE metA*5 *amyE1 ytsJ*1 *rsbV*37 *xre*1 *xkd*A1 *att*^SPβ^ *att*^ICE*Bs*1^); ^b^ BG1125 cells bearing pCB799-borne ε gene were grown in MMS7 containing 0.005% Xyl to titrate basal expression of the wt ζ toxin; ^c^ *E. coli* BL21(DE3) genotype (*ompT gal* (λ DE3, *int*::*lacI*-*Plac*_UV5_ T7 gene 1) *fhuA*2 (dcm) Δ*hsdS*).

When exponentially growing BG689 cells (~5 × 10^7^ cells/mL in MMS7) were transiently exposed to Fos or Amp (at twice the minimal inhibitory concentration [MIC]) or to Van (at 4 times the MIC) for 120 min, these bactericidal antimicrobials caused a cessation of cell proliferation, and rendered a fraction of 0.8 to 5 × 10^−3^ phenotypic tolerants (Figure 3). This multidrug tolerance of the isogenic population of antimicrobial sensitive cells is termed bacterial persistence [28,29,30].

To test whether toxin action depletes the UNAG pool and this leads to enhanced antimicrobial persistence when cells are treated with cell wall inhibitors moderate-density exponentially growing BG689 cells were treated with Xyl (to induce ζY83C toxin expression) and transiently exposed to the antimicrobial. Experiments are showed when the two compounds (*i.e*., Xyl and the antimicrobial) were added simultaneously, but the same results were obtained when first cells were exposed to toxin action for 30 min, and later the antimicrobial was added. Toxin ζY83C expression and Fos, Van or Amp treatment additively decreased the rate of cell survivals to 0.9 to 2 × 10^−6^ (Figure 3). Since the three antimicrobial show a similar outcome in the presence of free ζY83C toxin, it is unlike that the enhanced sensitivity could be attributed to a perturbation of cell envelop stress (see above). It is likely that: (I) ζ toxin expression only decreases the UNAG pool, and Fos, Van or Amp use the remaining fraction to further inhibit the plating of toxin tolerant cells; and (II) ζ toxin expression additively enhances the efficacy of the antimicrobials rather than making cells insensitive to Fos, Van or Amp treatment. Alternatively, transient expression of ζ toxin, by inactivating the UNAG substrate, inhibits the action of the MurA-MurF enzymes, and this inhibition leads to loss of cell shape and integrity, with cells becoming more prone to autolysis, when they are additionally treated with any of these antimicrobial.

### 2.4. Prolonged Action of Toxin ζY83C Does not Induce Massive Cell Lysis

To test the latter hypothesis were analyzed if prolonged expression of sub-physiological concentrations of ζY83C (360 or 480 min) might exhaust the metabolite pool and BG689 cells might reach the limits of their “capacity” to exit the dormant state, and they cannot be longer rescued from the “point of no return”. As already observed, after 16 h of ζY83C toxin expression the survival rate drops to 2–8 × 10^−7^ [16].

**Figure 4:**
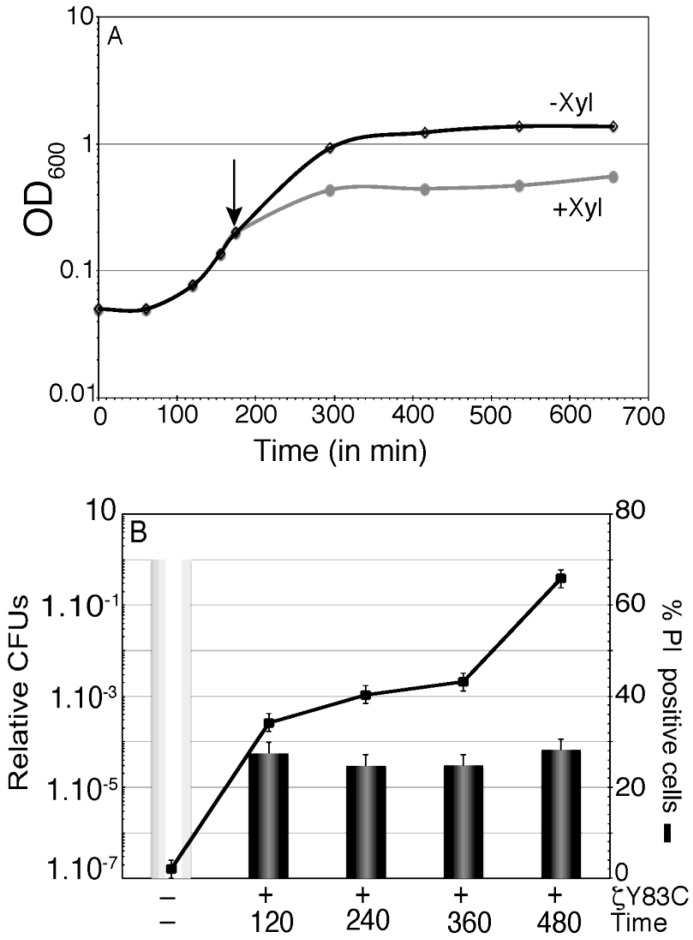
Effect of prolonged action of toxin ζY83C on membrane permeability and cell survival. (**A**) Growth curve of BG689 cells containing ζY83C gene. Cells were grown to ~2.5 × 10^7^ cell/mL in MMS7 (OD_600_ = 0.2), to half of the culture Xyl (0.5%) was added to induce ζY83C expression (time zero) and OD_600_ was followed over time in the two cultures. The arrow denotes the time of Xyl (0.5%) addition; (**B**) Aliquots of the cultures were taken at time zero, 120, 360 and 480 min after Xyl addition (denoted as +). The cells were fixed, stained with SYTO 9 and PI, and analyzed by fluorescence microscopy (black lane), at the same time, cells were washed and appropriate dilutions were plated in LB plates to count the survivals. The numbers of relative CFUs (black bars) at the indicated times after ζY83C toxin expression are relative to the non-induced control at the same time (denoted as -) taken as 1 (grey bar). Error bars show 95% confidence intervals of more than three independent experiments.

BG689 cells were grown up to optical density (OD_600_) of 0.2 in MMS7 and divided into two aliquots. To one aliquot inducer (0.5% Xyl) was added (time zero) and samples were collected at different times (120, 240, 360 and 480 min after induction) (Figure 4). In the presence of Xyl, the increment in OD_600_ halted after one doubling time, and remained constant during the 480 min interval (Figure 4A). At these time points, samples were withdrawn, and the cells were stained with membrane-permeant SYTO 9 (green fluorescence) and membrane-impermeant PI (red fluorescence), appropriate dilutions were plated in LB plates and the survival rate was measured (Figure 4B). Transient expression of ζY83C toxin for 120 min or longer periods of time induced dormancy, and a similar sub-fraction of 2–6 × 10^−5^ survivals became tolerant of toxin action (Figure 4B). Within the first 360 min of ζY83C toxin expression, the proportion of PI permeable cells slightly increased (~1.2-fold). At later times (480 min) the proportion of PI stained cells increased ~2-fold (~65% of total cells stained with PI) with respect to the proportion of cells stained with PI after 120 min of toxin expression (Figure 4B). It is likely that prolonged ζY83C expression might not induce massive cell lysis.

When similar experiments were performed in *E. coli* cells a different outcome is observed depending of the growth medium rather than the speed of growth [9,31]: In LB medium (doubling time ~28 min), 60 min after overexpression of PezTΔC242, cells underwent a massive death with few surviving cells showing an ovoid morphology [9]; in supplemented M9 medium (doubling time ~33 min), ζ toxin over-expression induced dormancy, and 60 min after induction massive filamentation, with subsequent growth recovery at later times was observed [31]. At present, the source of these discrepancies remains unknown.

### 2.5. Toxin ζ Reversibly Induces a Halt in Cell Proliferation

Previously, it was shown that transient exposure (120 min) to physiological concentrations of free wt ζ reversibly induces dormancy, produces a sub-fraction of membrane-compromised cells to be stained with PI (25%–35% of total cells), and a minor subpopulation of *B. subtilis* cells become tolerant of toxin action. Subsequent expression of the ε_2_ antitoxin facilitates the exit from the dormant state and a fraction of membrane compromised cells recover their alteration of the membrane potential [14].

Toxin ζ expression inactivated a fraction of UNAG and Fos used the remaining fraction of unphosphorylated UNAG to further inhibit cell proliferation (see Figure 3). If ζ toxin expression only reduces the UNAG pool and/or UNAG-P poisons the exit from the dormant state, expression of ε_2_ antitoxin might lead to different outcomes in the presence of Fos: First, if ζ toxin expression and Fos addition deplete the UNAG pool, cells should not be recovered by antitoxin expression and cell wall biosynthesis can be rescued only after *de novo* synthesis of UNAG or UNAG-3P has to be re-activated by an unknown pathway. Second, the concert action of both ζ toxin expression and Fos addition triggers cell lysis by unbalancing the control of peptidoglycan biosynthesis. Finally, if ζ toxin expression decreases a fraction of the UNAG pool, without depleting it, ε_2_ antitoxin expression may reverse toxin action and the remaining UNAG is used to rescue cell proliferation. To discriminate between all these possible outcomes, cell survival and the proportion of PI stained cells were analyzed after ε_2_ antitoxin expression.

When exponentially growing BG1125 cells (Table 1) reached a moderate-density (~5 × 10^7^ cells/mL in MMS7) 1 mM IPTG was added to induce ζ toxin expression. As already observed, expression of nearly physiological wt ζ toxin concentrations for 120 min induced dormancy, a fraction of the cell population (~30%) was stained with PI, and ~1 × 10^−5^ survivals tolerant to toxin action were obtained (Table 2). Subsequent expression of the ε_2_ antitoxin, after 120 min of IPTG addition, by Xyl (0.5%) addition, led to exit of the dormant state with CFUs increasing ~5000-fold, and only a small fraction (~10%) of cells were stained with PI (Table 2) [14,15]. Alternatively, resumption of cell growth might dilute the relative proportion of PI stained cells up to 10% of total cells.

In the absence of IPTG, addition of Fos to moderate-density exponentially growing BG1125 cells for 120 min reduced plating efficiency of the cells to levels similar to the ones observed in the BG689 background (~3 × 10^−3^ survivals), and as expected for a bactericidal antimicrobial the proportion of cells permeable to PI increased (~70% of total cells) (Table 2). Expression of the ε_2_ antitoxin under these conditions did nor alter the observed outcome (~70% of total cells stained with PI, and ~3 × 10^−3^ survivals). When moderate-density exponentially growing BG1125 cells were exposed to toxin (by addition of 1 mM IPTG) and Fos action, the toxin markedly enhanced the efficacy of Fos treatment, but no additive effect on membrane permeation to PI was observed (Table 2). Then, transient expression of the ε_2_ antitoxin led to partial exit of the dormant state, which increased >2000-fold CFUs, but it failed to decrease the proportion of PI permeable cells (Table 2). It is likely that: (I) toxin expression enhances Fos sensitivity, and such effect does not seem to correlate with increased rate of cell lysis; (II) neither toxin expression nor Fos addition deplete the UNAG pool, because ε_2_ antitoxin expression facilitates the exit of the dormant state of a large fraction of cells; and (III) unregulated levels of murein synthesis, by transient toxin expression and Fos addition, do not induce massive cell autolysis. We propose that ζ toxin and Fos decrease the UNAG pool, but when ε_2_ antitoxin expression halts ζ toxin activity, by forming an inactive complex (ζε_2_ζ), the remaining active fraction of UNAG is processed by MurA enzymes and cells exit the ζ-induced dormant state with resumption of growth as expected for a toxin that is bacteriostatic in nature.

**Table 2:** Additive effect of ζ toxin on Fos action and ε_2_ reversion of ζ-induced dormancy.

Conditions ^a^	TA ^c^	% PI stained cells ^d^	CFUs ^e^
-	ζ^−^ ε^(+)^	2.6 (1250)	2.3 × 10^8^
+IPTG ^b^	ζ^+^ ε^(+)^	30 (1314)	3.8 × 10^3^
+IPTG + Xyl ^b^	ζ^+^ ε^+^	9.8 (1642)	2.2 × 10^7^
+Fos	ζ^−^ ε^(+)^	73 (1055)	6.7 × 10^5^
+Fos + Xyl ^b^	ζ^−^ ε^+^	70 (1432)	6.6 × 10^5^
+IPTG ^b^ + Fos	ζ^+^ ε^(+)^	75 (1247)	<1.0 × 10^2^
+IPTG + Fos + Xyl ^b^	ζ^+^ ε^+^	68 (1560)	2.3 × 10^5^

^a^ BG1125 (ζ^+^) cells bearing pCB799-borne ε gene were exponentially grown to ~5 × 10^7^ cells/mL in MMS7 containing 0.005% Xyl (denoted as ε^(+)^); ^b^ Then, IPTG (1 mM to express ζ toxin) or Fos (2 × MIC) or both agents simultaneously were added and the cells incubated for 120 min. In the conditions where Xyl (0.5%) was added at min 120 (to express wt ε_2_ antitoxin, denoted as ε^+^), the cells were further incubated for 30 min and CFUs were counted on LB plates containing also Xyl; ^c^ The presence or the absence of induction of ζ or ε gene is indicated with a + or –, respectively, and the (+) symbol denotes the low level of expression of the ε gene by addition of 0.005% Xyl necessary to titrate basal expression of the wt ζ toxin; ^d^ The percentage of cells stained with PI are indicated and number of cells analyzed are shown in parentheses; ^e^ CFUs/mL were measured by plating appropriate dilutions on LB or LB-Xyl (0.5% final concentration) plates. The results are the average of at least four independent experiments and are within a 10% standard error.

## 3. Experimental Section

### 3.1. Bacterial Strains, Media and Growth Conditions

The bacterial strains BG689 and BG1125 bearing pCB799 used in this study were previously reported [12] and are described in Table 1. Addition of low Xyl concentration (0.005%) to BG1125 bearing pCB799 allowed the synthesis of low but significant ε_2_ antitoxin levels, expressed from pCB799-borne ε gene, necessary to titrate the basal ζ toxin levels and to avoid genetic rearrangements [14]. BG1125 cells bearing pCB799-borne ε gene were grown in the presence of low levels of Xyl (0.005%) necessary to induce a basal expression of the ε_2_ antitoxin. Low levels of the ε_2_ antitoxin are necessary to titrate the ~40 ζ toxin monomers/CFUs that are produced from escapes of LacI repression in the BG1125 strain. Addition of 1 mM IPTG, increased ζ toxin to a plateau concentration of ~1,700 wt ζ monomers/CFU at ~30 min, and the steady-state level of ζ remained for at least 240 min [see 14,15]. This toxin concentration is comparable to the level of wt ζ toxin, when the gene is in its native context and transcribed from its native promoter (cells bearing pBT233-borne ωεζ operon that are neutralized by saturating ε_2_ antitoxin concentrations) [11].

Expression of the *xylR P*_xylA_ ζY83C *cat* cassette (BG689 strain) was induced by addition of 0.5% Xyl and expression of the *lacI P_hsp_*ζ *spc* cassette (BG1125 strain) by addition of 1 mM IPTG. The BG689 or BG1125 bearing pCB799 isogenic strains were grown to mid-exponential phase (~5 × 10^7^ cells/mL) at 37 °C in MMS7 supplemented with the required amino acid (methionine and tryptophan) at 50 µg/mL [12].

The antimicrobial, except Van, used a 4-times MIC, was added at twice MIC. Cells were taken after 120 min, washed with fresh pre-warmed media (to remove the inducer and/or antimicrobial) and appropriated dilutions were plated on LB agar plates containing glucose (which switches off ζY83C expression) or on LB agar plates containing or not Xyl to express the ε_2_ antitoxin (ζ toxin cassette). The survival rate, derived from the number of CFUs obtained in a given condition relative to the CFUs of the non-induced/non-antimicrobial treated control is documented.

The MIC of the different antimicrobials tested was estimated by exposing 1–3 × 10^6^ cells/mL for 16 h at 37 °C in MMS7 with shaking (240 rpm) to different concentrations of the antimicrobials employed, and is the mean of the minimum concentration that gave no growth in three independent experiments. In the absence of inducer, the presence of the ζY83C (BG689 strain) or ζ gene and pCB799-borne ε gene (BG1125) does not affect the MIC [15].

For ζ toxin purification the stop codon of the ζ gene was replaced by 6 His and a stop codon. This ζ variant shows the same phenotype that wt ζ when expressed in *B. subtilis* cells [16]. This ζ gene variant was non-clonable in *E. coli* cells under the transcriptional control of the T7 RNA polymerase-dependent promoter (*P*_T7_), but cloning of the ω and ε genes, under its native RNA polymerase σ^A^-dependent promoter (*P*_ω_), in opposite orientation, overcame the toxicity exerted by the ζ variant, leading to pCB920 (Table 1).

### 3.2. Fluorescence Microscopy

To determine the proportion of “membrane-compromised” cells, they were harvested by centrifugation, washed to remove toxin inducer and/or the antimicrobial, and were stained with SYTO 9, which stains all bacteria with green fluorescence, and PI, which stains membrane-compromised bacteria with red fluorescence, according to the manufacturer’s instructions (Molecular Probes, Leiden). Cells were visualized using a BX61 Olympus microscope and Olympus CCD DP70 camera, with the appropriate filters as described [12].

### 3.3. Protein Purification

Plasmid pCB920-borne ζ, under the control of promoter 10 of phage T7 (*P*_10_), and ε gene under the control of native *P*_ω_, was used to overexpress ζ in *E. coli* BL21(DE3) cells harboring pCB920. IPTG was added to induce the expression of T7 RNAP (*lacI lac*_UV5_ T7 gene 1), and 30 min later rifampicin was added to selectively block the expression of the ω and ε genes. After 120 min of ζ expression from rifampicin-resistant *P*_10_, and full decay of the ε_2_ antitoxin, the cells were harvested. The over-expressed long-living ζ toxin was purified in two steps as follows: cells were lysed using the French Press in buffer A (50 mM phosphate buffer pH 7.5, 100 mM NaCl, 5% glycerol), and soluble ζ protein was bound to a Ni-NTA column, and eluted using an imidazole gradient (2 to 75 mM). The fractions containing the ζ protein were diluted to 25 mM NaCl, and loaded in a Q Sepharose column. Protein ζ was eluted in buffer B (50 mM Tris-HCl pH 7.5, 5% glycerol) containing a gradient of NaCl (25 to 150 mM). The fractions containing the ζ protein were dialyzed against buffer C (50 mM Tris-HCl pH 7.5, 100 mM NaCl) containing 50% glycerol and stored at −20 °C.

### 3.4. Biochemical Assays

The ATPase or GTPase activity of ζ was measured by incubating increasing ATP or GTP concentrations (containing 10 nM [α-^32^P]-ATP) and a fixed amount of UNAG (2 mM) with purified toxin in buffer A (50 mM Tris-HCl pH 7.5; 50 mM NaCl; 1 mM MgCl_2_) at 30 °C. Then, TLCs of the radiolabeled nucleotides or sugar nucleotides were performed on polyethyleneimine-cellulose plates with 0.85 M KH_2_PO_4_ (pH 3.4) as the mobile phase as described [14] followed by autoradiography.

Quantitative production of UNAG-3P, degradation of ATP and accumulation of ADP was determined by Maldi-TOF analysis as described [32]. ATP and UNAG were incubated or not with ζ toxin in buffer B (10 mM Tris-HCl, pH 7.5, 50 mM NaCl, 1 mM MgCl_2_) for 30 min. Equal volume of the analyte solution (1 µL) was mixed with 1 mL of matrix solution (2,4,6 trihydroxyacetophenone or 2,3,6-trihydroxyacetophenone, 10 mg/mL in acetonitrile/water, 1:1 *v*/*v*) and 1 mL of ammonium citrate (50 mg/mL in water). Then 1 mL of the mixture was applied to the MALDI-TOF sample plate and air dried. The mass spectra were acquired using a Ultraflex III TOFTOF (BRUKER) mass spectrometer (equipped with a ND:YAG laser) operating in negative reflector mode.

## 4. Conclusions

Toxin ζ-mediated dormancy induction can be attributed to the reduction of the ATP and/or GTP pools, the increment of the (p) ppGpp pool size and the accumulation of unreactive UNAG-3P among other changes (this work) [9]. We report here that purified ζ is mainly an UNAG-dependent ATPase rather than a GTPase. Preliminary data suggested that ATP hydrolysis and accumulation of unreactive UNAG-3P are not strictly coupled reactions. Our results indicate that physiological ζ toxin concentrations enhance the efficacy of antimicrobials that inhibit cell wall biosynthesis (Figure 3). A similar ζ-mediated sensitization to other three different classes of antimicrobials was reported previously [15].

Our results are incompatible with the hypothesis that ζ or PezT depletes the UNAG pool to generate an irreversible positive feedback loop, and as the cell wall blockage takes effect, cell wall weakening should instigate a suicide program and autolysis [9]. We surmise that cells can readjust their metabolism during situations where ζ toxin activation is temporary and reversible. Our *in vivo* experiments suggest that transient ζ toxin expression (120 min) reversibly induces a dormant state, leads to a sub-fraction (25%–35%) of the population stained with PI, and a minor subpopulation of cells exhibits non-inheritable toxin tolerance. Similarly, addition of Fos triggers stasis, leads to a fraction (65%–75%) of PI stained cells (suggesting cell death), and a minor subpopulation of cells are stochastically tolerant to Fos action. After ζ toxin expression and Fos addition, subsequent expression of the ε_2_ antitoxin specifically reverses ζ-induced dormancy, but it fails to reduce the proportion of Fos-induced PI stained cells.

From the results obtained in this work and previous results, we propose that the *modus operandi* of ζ toxin is to induce diverse responses to cope with stress, and reduction of the UNAG pool is one among them, rather than instigating a suicide program by depleting UNAG. A similar scenario is observed upon long time ζ toxin accumulation (360 or 480 min). Here, tolerant cells might enter into the viable-but-nonculturable state, but massive cell lysis was not observed. Conversely, after 60 min over-expression in *E. coli* cells, the PezTΔC242 toxin blocks cell wall synthesis and triggers massive autolysis with few intact cells with an ovoid morphology [9], but such massive autolysis was not observed upon ζ toxin over-expression [31]. It is likely that the truncated and attenuated version of the PezT toxin (PezTΔC242) is bactericidal rather than bacteriostatic in nature.
